# Clinical course and management of children with IgA vasculitis with nephritis

**DOI:** 10.1007/s00467-023-06023-8

**Published:** 2023-06-14

**Authors:** Hillarey K. Stone, Mark Mitsnefes, Kimberley Dickinson, Evanette K. Burrows, Hanieh Razzaghi, Ingrid Y. Luna, Caroline A. Gluck, Bradley P. Dixon, Vikas R. Dharnidharka, William E. Smoyer, Michael J. Somers, Joseph T. Flynn, Susan L. Furth, Charles Bailey, Christopher B. Forrest, Michelle Denburg, Edward Nehus

**Affiliations:** 1 Division of Nephrology and Hypertension, Cincinnati Children’s Hospital Medical Center, 3333 Burnet Avenue, MLC 7022, Cincinnati, OH 45229 USA; 2 Department of Pediatrics, University of Cincinnati College of Medicine, Cincinnati, OH USA; 3 Applied Clinical Research Center, Children’s Hospital of Philadelphia, Philadelphia, PA USA; 4 Department of Pediatrics, Children’s Hospital of Philadelphia, Philadelphia, PA USA; 5 Department of Biomedical and Health Informatics, Children’s Hospital of Philadelphia, Philadelphia, PA USA; 6 Division of Pediatric Nephrology, Alfred I. duPont Hospital for Children, Wilmington, DE USA; 7 Renal Section, Department of Pediatrics, University of Colorado School of Medicine, Aurora, CO USA; 8 Division of Pediatric Nephrology, Washington University School of Medicine, Saint Louis, MO USA; 9 Center for Clinical and Translational Research, Abigail Wexner Research Institute, Nationwide Children’s Hospital, The Ohio State University College of Medicine, Columbus, OH USA; 10 Division of Nephrology, Department of Medicine, Boston Children’s Hospital, Harvard Medical School, Boston, MA USA; 11 Division of Nephrology, Department of Pediatrics, Seattle Children’s Hospital and University of Washington School of Medicine, Seattle, WA USA; 12 Department of Epidemiology, Perelman School of Medicine at the University of Pennsylvania, Philadelphia, PA USA; 13 Division of Nephrology, Children’s Hospital of Philadelphia, Philadelphia, PA USA; 14 Department of Pediatrics, West Virginia University Charleston Campus, Charleston, WV USA

**Keywords:** Nephrology, Vasculitis, Glomerulonephritis, Chronic kidney disease, Henoch-Schönlein purpura

## Abstract

**Background:**

IgA vasculitis is the most common vasculitis in children and is often complicated by acute nephritis (IgAVN). Risk of chronic kidney disease (CKD) among children with IgAVN remains unknown. This study aimed to describe the clinical management and kidney outcomes in a large cohort of children with IgAVN.

**Methods:**

This observational cohort study used the PEDSnet database to identify children diagnosed with IgAV between January 1, 2009, and February 29, 2020. Demographic and clinical characteristics were compared among children with and without kidney involvement. For children followed by nephrology, clinical course, and management patterns were described. Patients were divided into four categories based on treatment: observation, renin–angiotensin–aldosterone system (RAAS) blockade, corticosteroids, and other immunosuppression, and outcomes were compared among these groups.

**Results:**

A total of 6802 children had a diagnosis of IgAV, of whom 1139 (16.7%) were followed by nephrology for at least 2 visits over a median follow-up period of 1.7 years [0.4,4.2]. Conservative management was the most predominant practice pattern, consisting of observation in 57% and RAAS blockade in 6%. Steroid monotherapy was used in 29% and other immunosuppression regimens in 8%. Children receiving immunosuppression had higher rates of proteinuria and hypertension compared to those managed with observation (*p* < 0.001). At the end of follow-up, 2.6 and 0.5% developed CKD and kidney failure, respectively.

**Conclusions:**

Kidney outcomes over a limited follow-up period were favorable in a large cohort of children with IgAV. Immunosuppressive medications were used in those with more severe presentations and may have contributed to improved outcomes.

**Graphical abstract:**

**Figure:**
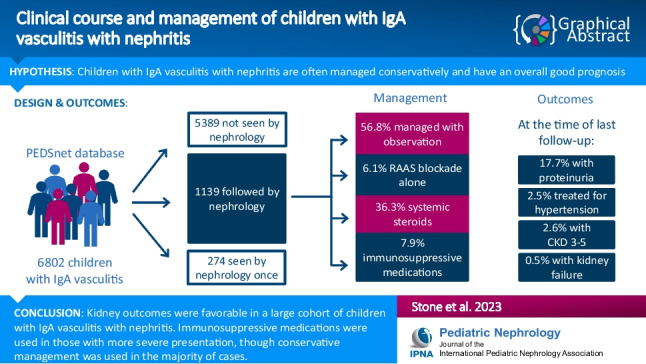
A higher resolution version of the Graphical abstract is available as Supplementary information

**Supplementary Information:**

The online version contains supplementary material available at 10.1007/s00467-023-06023-8.

## Introduction

IgA vasculitis (IgAV) (formerly Henoch-Schönlein purpura or HSP) is the most common vasculitis seen in children, with an incidence of 6–20 cases per 100,000 children per year [1]. IgAV affects small vessels and presents with palpable purpuric rash, arthritis or arthralgia, and/or abdominal pain. Approximately 5–30% of patients with IgAV develop nephritis (IgAVN), and long-term prognosis primarily depends upon the extent of kidney involvement [1, 2]. IgAVN has been historically considered a self-limited condition; however, it is now recognized that a subset of patients develop CKD [3–5].

Several studies have assessed outcomes in patients with IgAVN, with rates of CKD ranging from 1.6 to 44% [2, 3, 6]. These have largely been single-center studies limited by small sample sizes. Therefore, the risk of CKD in children with IgAVN remains unclear. Accurate prediction of CKD risk in children with IgAVN is an essential component of developing treatment strategies. Several small studies have reported the use of various immunosuppressive regimens, including corticosteroids, cyclophosphamide, cyclosporine, and azathioprine [7–11]. In the absence of specific evidence-based guidelines, the optimal clinical management of IgAVN remains uncertain. Therefore, we hypothesized that wide variation would exist in the management of children with IgAVN.

The current study aimed to describe the clinical management and outcomes of children with IgAVN using a large representative sample of children followed by nephrology. To accomplish this, we used data from PEDSnet, a national clinical research network comprised of 7 pediatric health systems [12]. We compared clinical characteristics and outcomes among children managed conservatively and those receiving immunosuppressive medications.

## Methods

### Study setting and data source

Electronic health record (EHR) data were obtained from PEDSnet (pedsnet.org), a national network of pediatric health systems. Specific institutions included: Children’s Hospital of Philadelphia, Cincinnati Children’s Hospital Medical Center, Children’s Hospital Colorado, Nationwide Children’s Hospital, Nemours Children’s Health System, Seattle Children’s Hospital, and St. Louis Children’s Hospital. EHR data were shared for all healthcare encounters provided in outpatient, inpatient, and emergency department settings at these institutions.

PEDSnet uses EHR data in a common data model to assemble a longitudinal observational data resource. EHR records for all children seen at a member institution since 2009 are extracted, transformed to a common data format, and merged into the data resource quarterly. Data for this study were from PEDSnet database version 3.7 (extracted in March 2020). Visit diagnoses and problem list entries assigned by clinicians using intelligent medical objects (IMO) clinical interface terminology and diagnosis codes from billing sources are standardized to Systematized Nomenclature of Medicine, Clinical Terms (SNOMED-CT) [13]. The institutional review boards (IRB) from each of the contributing institutions reviewed the study and deemed it nonhuman subject research/exempt from IRB review.

### Patients

Using SNOMED-CT codes, unique patients with a diagnosis of IgAV/HSP seen at a PEDSnet institution between January 1, 2009, and February 29, 2020, were included (*n* = 7245). Exclusion criteria included diagnosis of another vasculitis (*n* = 32) or IgA nephropathy (*n* = 46). A full list of codes used for inclusion and exclusion criteria and code lists used to derive clinical variables are available at https://github.com/PEDSnet/IgAVN. To focus on outcomes related to IgAV and not attributable to other chronic conditions, we used the Pediatric Medical Complexity Algorithm (PMCA) [14] to stratify children into one of three categories: complex chronic disease, noncomplex chronic disease, and without chronic disease. Using this system, we excluded patients with a nonrenal progressive or malignant condition (*n* = 365) [14].

### Identification of kidney involvement

Contact with nephrology was used to identify patients with significant kidney involvement, as stratification based on diagnosis code and/or laboratory findings was noted to miss a large number of patients with apparent kidney involvement (i.e., who were followed by nephrology, managed with pharmacologic agents, who underwent kidney biopsy, etc.). Patients were divided into three groups based on contact with nephrology: patients never seen by nephrology (group 0), seen by nephrology once (group 1), and followed by nephrology ≥ 2 times (group 2). Demographic and baseline clinical data were summarized and compared among patients in these three groups.

### Clinical variable definitions

Several clinical measures were evaluated to assess outcomes in children with IgAV/HSP. The bedside Schwartz equation [15] was used to derive estimated glomerular filtration rate (eGFR). Proteinuria was defined as ≥ 2 + protein on urinalysis. Hematuria was defined by a qualitative dipstick result (e.g., positive and moderate) or a quantitative microscopic result of > 5 red blood cells/high power field. Urine protein to creatinine ratio (UPCR) was reported as a ratio (mg:mg) where available or calculated using a urine protein and urine creatinine value from the same date and then categorized as < 0.5 (non-significant proteinuria), 0.5–2.0 (significant proteinuria), and > 2.0 (nephrotic range proteinuria). Hypoalbuminemia was defined as serum albumin of < 2.5 g/dL. Advanced CKD (stage ≥ 3) was defined as an eGFR less than 60 mL/min/1.73 m^2^ on two separate occasions ≥ 3 months apart, with no eGFR above 90 mL/min/1.73 m^2^ between or after these values, except when a patient received a kidney transplant. Using normative data from the 2017 clinical practice guideline [16], uncontrolled systolic blood pressure (SBP) was defined as SBP ≥ 95th percentile for children under 13 years or SBP ≥ 130 mmHg for patients ≥ 13 years. Similarly, uncontrolled diastolic blood pressure (DBP) was defined as DBP ≥ 95th percentile for children under 13 years or DBP ≥ 80 mmHg for patients ≥ 13 years. Dialysis was defined using a list of procedure codes, which were designated by the study team as chronic or not chronic. Chronic dialysis was defined by at least one chronic dialysis code or two non-chronic dialysis codes separated by at least 90 days. Kidney failure (KF) was defined by outcomes of chronic dialysis or kidney transplant.

Demographic data were collected from the date of cohort entrance. Laboratory values and other clinical data were assessed from the period of initial assessment (baseline period, defined as 30 days before through 90 days after the initial IgAV/HSP diagnosis) and at the time of final follow-up. For the baseline period, the height and serum creatinine value closest to the date of initial diagnosis were used to calculate eGFR. Proteinuria and hematuria were reported if present at any point during the baseline period. The highest UPCR and lowest serum albumin values available during the baseline period were reported. Height, weight, SBP, DBP, and BMI values closest to the date of initial diagnosis were used, averaging same-day measurements recorded on the closest date when necessary. Of note, lab data was unavailable for one site. These numbers are reported in each table’s legend. All other data (including demographic data, medication use, etc.) were available for this site.

Follow-up time was defined as time from initial IgAV/HSP diagnosis through final contact in the PEDSnet network, or through the date of the first of either chronic dialysis, transplant, or death.

Among patients with at least two nephrology contacts, clinical course and outcomes were described. Height and serum creatinine closest to the final contact were used to calculate eGFR. UPCR was derived using values closest to the final contact. Proteinuria, hematuria, and hypoalbuminemia were reported in the follow-up period if occurring at the last available measurement. KF was reported if patients met criteria at any time during follow-up.

### Statistical analysis

Categorical variables were expressed using frequency and percentage, while continuous variables were reported using median and interquartile range. Continuous variables were compared using the Wilcoxon rank-sum test and categorical variables with the chi-square test. Given large sample sizes, baseline data were also compared using standard mean difference (SMD). Statistical analyses were performed using R software version 4.0.3.

## Results

### Cohort baseline characteristics

Among 6,753,739 patients in the PEDSnet database, we identified 6802 (0.1%) with IgAV/HSP meeting selection criteria. Of those, 53.4% were male, 72.4% were White/Caucasian, 6.0% were Black, 3.6% were Asian, and 12.5% were Hispanic. Compared with all patients in the PEDSnet database, the IgAV cohort contained a significantly lower percentage of Black patients (6.0% vs. 16.3%, *p* < 0.001). Among all patients with IgAV/HSP, 274 (4.0%) were seen by nephrology once, and 1139 (16.7%) were followed by nephrology more than once (Table 1). Kidney involvement (defined as hematuria and/or proteinuria during the baseline period) was present in 29% of those with available laboratory data.

**Table 1:** Baseline characteristics for all patients with IgAV/HSP. Categorical variables are expressed as frequency (percentage), and continuous variables are expressed as median [IQR]. P values and standard mean difference (SMD) compare group 0 vs. group 2, and then groups 1 vs. group 2. Of note, lab data were missing for one site but were available for 4842 patients in group 0, 253 patients in group 1, and 1001 patients in group 2

	Full PEDSnet Database	Full IgAV cohort *n* = 6802	Group 0 (Never seen by nephrology) *n* = 5389	Group 1 (1 nephrology visit) *n* = 274	Group 2 (2 or more nephrology visits) *n* = 1139	*P*-value (Group 0 vs. group 2, group 1 vs. group 2)	SMD (Group 0 vs. group 2, group 1 vs. group 2)
Demographic data							
Male	(51.0)	3632 (53.4)	2884 (53.5)	142 (51.8)	603 (52.9)	(0.75, 0.79)	(0.01, 0.02)
Age (years)		6.61	6.25 [4.33, 8.83]	7.59 [5.57, 10.67]	8.09 [5.81, 11.67]	(< 0.001, 0.04)	(0.47, 0.16)
Age category						(< 0.001, 0.02)	(0.50, 0.20)
< 2		245 (3.6)	231 (4.3)	7 (2.6)	7 (0.6)		
2–4		1806 (26.6)	1572 (29.2)	52 (19.0)	182 (16.0)		
5–9		3267 (48.0)	2582 (47.9)	135 (49.3)	550 (48.3)		
10–14		1032 (15.2)	699 (13.0)	56 (20.4)	277 (24.3)		
≥ 15		452 (6.6)	305 (5.7)	24 (8.8)	123 (10.8)		
Age ≥ 8		2392 (35.2)	1688 (31.3)	124 (45.3)	580 (50.9)	(< 0.001, 0.11)	(0.41, 0.11)
Race						(0.02, 0.99)	(0.11, 0.02)
White/Caucasian	(58.2)	4926 (72.4)	3883 (72.1)	204 (74.5)	839 (73.7)		
Black/African American	(16.3)	408 (6.0)	350 (6.5)	11 (4.0)	47 (4.1)		
Asian	(3.4)	245 (3.6)	200 (3.7)	8 (2.9)	37 (3.2)		
Other/Unknown	(22.1)	1223 (18.0)	956 (17.7)	51 (18.6)	216 (19.0)		
Ethnicity						(0.14, 0.11)	(0.07, 0.13)
Hispanic	(12.2)	851 (12.5)	672 (12.5)	33 (12.0)	146 (12.8)		
Not Hispanic	(74.6)	5511 (81.0)	4358 (80.9)	218 (79.6)	935 (82.1)		
Other/Unknown	(13.2)	440 (6.5)	359 (6.7)	23 (8.4)	58 (5.1)		
Baseline clinical data							
BMI percentile	68%ile [35, 91]		62%ile [30, 86]	72%ile [38, 91]	68%ile [38, 90]	(< 0.001, 0.53)	(0.17, 0.04)
BMI ≥ 85%ile		1044 (15.3)	631 (26.6)	74 (34.7)	339 (32.6)	(< 0.001, 0.61)	(0.13, 0.05)
BMI ≥ 95%ile		519 (7.6)	306 (12.9)	36 (16.9)	177 (17.0)	(0.002, 1.00)	(0.12, 0.004)
SBP percentile			81%ile [58, 95]	78%ile [56, 95]	80%ile [55, 94]	(0.17, 0.94)	(0.06, 0.03)
DBP percentile			77%ile [58, 91]	73%ile [51, 87]	70%ile [49, 88]	(< 0.001, 0.78)	(0.24, 0.02)
Systolic hypertension		1330 (19.6)	1032 (19.2)	55 (20.1)	243 (21.3)	(0.10, 0.71)	(0.05, 0.03)
Diastolic hypertension		961 (14.1)	769 (14.3)	28 (10.2)	164 (14.4)	(0.95, 0.09)	(0.004, 0.13)
Hypertension diagnosis		343 (5.0)	115 (2.1)	25 (9.1)	203 (17.8)	(< 0.001, 0.001)	(0.54, 0.26)
eGFR							
eGFR available			1385 (28.6)	105 (41.5)	648 (64.7)		
eGFR (mL/min/1.73 m^2^)			123.0 [105.7,142. 7]	114.3 [99.3,143.3]	115.1 [96.3,133.3]	(< 0.001,0.37)	0.31, 0.11
eGFR category						(< 0.001,0.21)	0.31, 0.24
≥ 90		1904 (89.1)	1275 (92.1)	92 (87.6)	537 (82.9)		
≥ 60 and < 90		195 (9.1)	103 (7.4)	7 (6.7)	85 (13.1)		
≥ 30 and < 60		28 (1.3)	5 (0.4)	5 (4.8)	18 (2.8)		
< 30		11 (0.51)	2 (0.1)	1 (1.0)	8 (1.2)		
Serum albumin							
Serum albumin available			1545 (31.9)	94 (37.2)	571 (57.0)		
Median ([IQR]			4.00 [3.60, 4.30]	4.00 [3.50, 4.30]	4.00 [3.50, 4.30]	(0.11, 0.90)	0.13, 0.08
Hypoalbuminemia		57 (2.6)	26 (1.7)	8 (8.5)	23 (4.0)	(0.003, 0.10)	0.14, 0.19
Proteinuria							
Urine protein available			3154 (65.1)	179 (70.8)	856 (85.5)		
≥ 2 + on UA		629 (15.0)	196 (6.2)	47 (26.3)	386 (45.1)	(< 0.001, < 0.001)	1.00, 0.40
UPCR available			207 (4.3)	64 (25.3)	442 (44.2)		
UPCR			0.19 [0.10, 0.50]	0.30 [0.12, 1.13]	0.82 [0.25, 2.72]	(< 0.001, < 0.001)	0.09, 0.26
UPCR category						(< 0.001, 0.01)	0.79, 0.43
< 0.5		370 (51.9)	155 (74.9)	37 (57.8)	178 (40.3)		
0.5–2		186 (26.1)	36 (17.4)	18 (28.1)	132 (29.9)		
> 2		157 (22.0)	16 (7.7)	9 (14.1)	132 (29.9)		
Microscopic hematuria							
Urine blood available			3565 (73.6)	180 (71.1)	859 (85.8)		
Microscopic hematuria		1101 (23.9)	451 (12.7)	79 (43.9)	571 (66.5)	(< 0.001, < 0.001)	1.32, 0.47
Urine blood and urine protein available			3151 (65.1)	177 (70.0)	850 (84.9)		
Proteinuria (≥ 2 +) and Hematuria		517 (12.4)	123 (3.9)	42 (23.7)	352 (41.4)	(< 0.001, < 0.001)	1.00, 0.39

*BMI*, body mass index; *SBP*, systolic blood pressure; *DBP*, diastolic blood pressure; *eGFR*, estimated glomerular filtration rate; *UA*, urinalysis; *UPCR*, urine protein to creatinine ratio

Table 1 includes demographic and baseline clinical data for all children in the IgAV cohort, then compares clinical characteristics of patients followed by nephrology (group 2) with those who did not see (group 0) or only had one visit with nephrology (group 1). Average age at diagnosis for the full IgAV cohort was 6.6 years, and those followed by nephrology were significantly older, with a median age at diagnosis of 8.1 years (*p* < 0.001, SMD 0.47). They were also significantly more likely to have proteinuria, hematuria, lower eGFR, and a diagnosis of hypertension at baseline (all SMD > 0.3).

### Management of patients followed by nephrology

Based on data described in Table 1, we identified that patients followed by nephrology two or more times had the most significant kidney involvement. Therefore, the remainder of our analyses focused on patients in this group. A total of 1139 patients (16.7% of the study sample) were seen by nephrology at least twice. As described in Table 2, kidney biopsy was performed in 166 patients (14.6%); 13.6% were also seen by gastroenterology and 26.4% by rheumatology in the first year after diagnosis. The majority of patients (56.8%) were managed with supportive care or observation alone (without renin–angiotensin–aldosterone system (RAAS) blockade or immunosuppressive agents). Among those receiving pharmacological therapy, 16.4% were treated with RAAS blockade, including 6.1% managed with RAAS blockade alone. A total of 36.3% received systemic corticosteroids, and 7.9% received another immunosuppressive agent (with or without corticosteroids). Azathioprine and mycophenolate mofetil (MMF) were most frequently used and prescribed in 53.3% and 42.2% of patients receiving immunosuppressive medications, respectively. Eighteen percent were diagnosed with hypertension during the first year after diagnosis. Anti-hypertensive medications other than RAAS blockade were used in 9.4%, the most common being calcium channel blockers.

**Table 2:** Clinical course in the first year after diagnosis for patients followed by nephrology (group 2). Categorical variables are expressed as frequency (percentage), and continuous variables are expressed as median [IQR]

Time to first nephrology visit (days)	0.0 [0.0, 29.0]
Kidney biopsy	166 (14.6)
Seen by GI	155 (13.6)
Seen by rheumatology	301 (26.4)
Treatment	
Observation	647 (56.8)
RAAS blockade alone	69 (6.1)
RAAS blockade overall	187 (16.4)
Systemic Corticosteroids (all)	414 (36.3)
Enteral corticosteroids	400 (35.1)
IV corticosteroids	137 (12.0)
Systemic corticosteroids > 1 prescription	316 (27.7)
Immunosuppressive medication	90 (7.9)
Azathioprine	48 (4.2)
Cyclophosphamide	8 (0.7)
Calcineurin inhibitor (CNI)	6 (0.5)
Mycophenolate (MMF)	38 (3.3)
Rituximab	3 (0.3)
Hypertension diagnosis	244 (21.4)
Treated with RAAS blocker	187 (16.4)
Treated with other antihypertensive	107 (9.4)
Calcium channel blocker	73 (6.4)
Beta blocker	16 (1.4)
Diuretic (loop and thiazide)	54 (4.7)
Other antihypertensive medication	54 (4.7)

*GI*, gastroenterology; *RAAS*, renin–angiotensin–aldosterone system; *IV*, intravenous

Table 3 compares demographic and baseline clinical factors stratified by treatment group: observation alone, RAAS blockade alone, corticosteroids, and other immunosuppressive medications. Median age at diagnosis was significantly higher in the RAAS blockade and immunosuppression groups. Patients treated with corticosteroids and/or immunosuppressive medications had significantly lower serum albumin, higher UPCR, more hematuria, and more hypertension at baseline than patients in the conservative management (observation alone or RAAS blockade alone) groups. Median eGFR at baseline was above 100 mL/min/1.73 m^2^ for all groups. The percentage of patients with eGFR less than 90 mL/min/1.73 m^2^ at baseline was higher in the RAAS blockade (37.2%) and immunosuppression (27.9%) groups compared with the observation (14.7%) and corticosteroid (14.1%) groups.

**Table 3:** Demographic and baseline clinical characteristics for patients followed by nephrology (group 2), stratified by management group (observation alone, RAAS blockade alone, corticosteroids with or without RAAS blockade, immunosuppressive medications with or without corticosteroids and/or RAAS blockade). Categorical variables are expressed as frequency (percentage), and continuous variables are expressed as median [IQR]. Of note, lab data were missing for one site but were available for 572 patients in the observation alone group, 57 in the RAAS blockade alone group, 292 in the steroids group, and 80 in the immunosuppression group

	Observation (*n* = 647)	RAAS Blockade alone (*n* = 69)	Steroids (*n* = 333)	Other Immunosuppression (*n* = 90)	*P*-value
Male	329 (50.9)	34 (49.3)	194 (58.3)	46 (51.1)	0.14
Age (years)	7.9 [5.7, 11.5]	11.1 [8.0, 15.4]	7.7 [5.6, 10.8]	9.4 [7.0, 13.4]	< 0.001
Year of first diagnosis					0.001
< 2011	140 (21.6)	26 (37.7)	49 (14.7)	16 (17.8)	
2011–2013	157 (24.3)	14 (20.3)	81 (24.3)	20 (22.2)	
2014–2016	144 (22.3)	15 (21.7)	103 (30.9)	21 (23.3)	
≥ 2017	206 (31.8)	14 (20.3)	100 (30.0)	33 (36.7)	
Baseline eGFR	113.2 [98.4, 130.6]	103.5 [84.3, 118.4]	121.84 [102.7, 139.5]	104.2 [89.7, 125.3]	< 0.001
Baseline eGFR category					< 0.001
≥ 90	254 (85.2)	22 (62.9)	213 (85.9)	49 (72.1)	
≥ 60 and < 90	31 (10.4)	12 (34.3)	30 (12.1)	12 (17.6)	
≥ 30 and < 60	9 (3.0)	0 (0.0)	3 (1.2)	6 (8.8)	
< 30	4 (1.3)	1 (2.9)	2 (0.8)	1 (1.5)	
Baseline serum albumin	4.1 [3.7, 4.4]	4.0 [3.7, 4.4]	3.8 [3.3, 4.3]	3.5 [3.0, 4.0]	< 0.001
Hypoalbuminemia at baseline	3 (1.2)	1 (3.3)	9 (4.0)	10 (15.9)	< 0.001
Baseline UPCR	0.39 [0.15, 1.17]	0.84 [0.31, 1.67]	1.02 [0.28, 3.34]	4.05 [1.38, 8.35]	< 0.001
Baseline UPCR category					< 0.001
< 0.5	106 (55.5)	12 (37.5)	54 (35.1)	6 (9.2)	
0.5–2	57 (29.8)	14 (43.8)	47 (30.5)	14 (21.5)	
> 2	28 (14.7)	6 (18.8)	53 (34.4)	45 (69.2)	
Baseline urine blood available	474 (82.9)	43 (75.4)	271 (92.8)	71 (88.8)	
Hematuria at baseline	268 (56.5)	33 (76.7)	202 (74.5)	68 (95.8)	< 0.001
Baseline urine protein available	472 (82.5)	42 (73.7)	272 (93.2)	70 (87.5)	
Proteinuria at baseline (≥ 2 +)	147 (31.1)	32 (76.2)	145 (53.3)	62 (88.6)	< 0.001
Baseline urine protein and baseline urine blood available	467 (81.6)	42 (73.7)	271 (92.8)	70 (87.5)	
Proteinuria (≥ 2 +) and hematuria during baseline	128 (27.4)	27 (64.3)	135 (49.8)	62 (88.6)	< 0.001
Baseline hypertension (diagnosis)	63 (9.7)	18 (26.1)	82 (24.6)	40 (44.4)	< 0.001
Baseline systolic hypertension	94 (14.5)	18 (26.1)	104 (31.2)	27 (30.0)	< 0.001
Baseline diastolic hypertension	60 (9.3)	6 (8.7)	79 (23.7)	19 (21.1)	< 0.001

*RAAS*, renin–angiotensin–aldosterone system; *eGFR*, estimated glomerular filtration rate; *UPCR*, urine protein to creatinine ratio

### Outcomes at last follow-up

Median follow-up time was 1.7 years [0.4,4.2], and patients were seen by nephrology a median of 7 times [IQR 3,15] during the follow-up period. Of those patients with follow-up data available, 2.6% had CKD stage ≥ 3, and 17.7% had a UPCR ≥ 0.5. Antihypertensive medications were prescribed in 2.5%, and 6.6% of patients had a diagnosis of hypertension (Table 4). Two patients required dialysis, and one received a kidney transplant during the follow-up period. No deaths occurred during the follow-up period, though one patient died during the baseline period.

**Table 4:** Clinical outcomes at the time of last follow-up for patients followed by nephrology. Categorical variables are expressed as frequency (percentage), and continuous variables are expressed as median [IQR]. Of note, lab data were missing for one site but were available for 543 patients followed < 4 years and 270 patients followed ≥ 4 years

	All (*n* = 921)	Patients followed < 4 years (*n* = 616)	Patients followed ≥ 4 years (*n* = 305)	*P*-value
Hypertension diagnosis within 6 months prior to final encounter	61 (6.6)	37 (6.0)	24 (7.9)	0.35
Treated with antihypertensive within 6 months prior to final encounter	23 (2.5)	15 (2.4)	8 (2.6)	1.00
Calcium channel blocker	10 (1.1)	7 (1.1)	3 (1.0)	1.00
Beta blocker	4 (0.4)	3 (0.5)	1 (0.3)	1.00
Diuretic (loop and thiazide)	5 (0.4)	4 (0.6)	1 (0.3)	0.88
Other antihypertensive medication	8 (0.9)	4 (0.6)	4 (1.3)	0.52
RAAS blockade overall within 6 months prior to final encounter	62 (6.7)	39 (6.3)	23 (7.5)	0.58
eGFR at last measurement				
eGFR available	388 (47.7)	216 (39.8)	172 (63.7)	
eGFR (mL/min/1.73 m^2^)	110.4 [92.3, 128.5]	113.3 [96.5, 132.1]	106.0 [92.0, 124.6]	0.03
eGFR category				0.74
≥ 90	304 (78.4)	171 (79.2)	133 (77.3)	
≥ 60 and < 90	71 (18.3)	39 (18.1)	32 (18.6)	
≥ 30 and < 60	7 (1.8)	4 (1.9)	3 (1.7)	
< 30	6 (1.5)	2 (0.9)	4 (2.3)	
Serum albumin available	354 (43.5)	190 (35.0)	164 (60.7)	
Serum albumin	4.3 [4.0, 4.6]	4.3 [4.0, 4.6]	4.3 [4.0, 4.6]	0.96
Hypoalbuminemia (Serum albumin < 2.5)	2 (0.6)	2 (1.1)	0 (0.0)	0.54
Proteinuria				
Urine protein available	606 (74.5)	391 (72.0)	215 (79.6)	
≥ 2 + on UA	78 (12.9)	49 (12.5)	29 (13.5)	0.83
UPCR at last measurement				
UPCR available	317 (39.0)	208 (38.3)	109 (40.4)	
UPCR	0.16 [0.08, 0.35]	0.16 [0.08, 0.36]	0.17 [0.10, 0.33]	0.28
UPCR category				0.61
< 0.5	261 (82.3)	169 (81.2)	92 (84.4)	
0.5–2	43 (13.6)	31 (14.9)	12 (11.0)	
> 2	13 (4.1)	8 (3.8)	5 (4.6)	
Hematuria at last measurement				
Urine blood available	606 (74.5)	390 (71.8)	216 (80.0)	
Microscopic hematuria	206 (34.0)	154 (39.5)	52 (24.1)	< 0.001
Proteinuria (≥ 2 +) and hematuria at last measurement	57 (9.4)	42 (10.7)	15 (7.0)	0.17
# of nephrology visits (per person-year)	3.8 [1.2, 9.1]	5.8 [2.5, 12.2]	1.3 [0.5, 3.2]	< 0.001
CKD stage ≥ 3*	10 (2.6)	2 (0.9)	8 (4.7)	0.05
eGFR or UPCR available	482 (59.3)	293 (54.0)	189 (70.0)	
eGFR AND UPCR available	223 (27.4)	131 (24.1)	92 (34.1)	
CKD ≥ 3 and/or UPCR ≥ 0.5	62 (12.9)	40 (13.7)	22 (11.6)	0.61
Dialysis	2 (0.2)	1 (0.2)	1 (0.3)	1.00
Chronic dialysis	1 (0.1)	0 (0.0)	1 (0.3)	0.72
Time to initiation of chronic dialysis (days)	1930 [1930, 1930]	N/A	1930 [1930, 1930]	
Kidney transplant	1 (0.1)	1 (0.2)	0 (0.0)	1.00
Time to kidney transplant (days)	308 [122, 436]	308 [122, 436]	NA	
Death	0	0	NA	NA
Time to death (days)	NA	NA	NA	NA
Chronic dialysis, transplant, and/or death	2 (0.2)	1 (0.2)	1 (0.3)	1.00

*RAAS*, renin–angiotensin–aldosterone system; *eGFR*, estimated glomerular filtration rate; *UA*, urinalysis; *UPCR*, urine protein to creatinine ratio; *CKD*, chronic kidney disease

Patients were further stratified based on total follow-up time of < 4 years versus ≥ 4 years in order to identify patterns attributable to duration of follow-up. There was no significant difference in the rate of proteinuria between the two groups. Patients followed for ≥ 4 years had a lower rate of hematuria (39.5% vs. 24.1%, *p* < 0.001) and an increased prevalence of CKD stage ≥ 3 (0.9 vs. 4.7%, *p* = 0.048).

A total of 113 patients had follow-up of ≥ 7 years. Of these, just two (1.8%) had CKD ≥ 3 at the time of last follow-up, and another seven (6.2%) had significant proteinuria with UPCR ≥ 0.5. No patients in the group followed ≥ 7 years experienced kidney failure or death.

Table 5 compares outcomes for patients followed by nephrology managed with observation, RAAS blockade alone, corticosteroids, or other immunosuppressive agents. Proteinuria and hypertension were more common in patients managed with RAAS blockade or immunosuppressive therapy; however, eGFR and rates of advanced CKD at the end of follow-up were not significantly different among patients in the four treatment groups.

**Table 5:** Clinical outcomes at the time of last follow-up for patients followed by nephrology, stratified by treatment group. Categorical variables are expressed as frequency (percentage), and continuous variables are expressed as median [IQR]. Of note, lab data were missing for one site but were available for 418 patients in the observation alone group, 52 in the RAAS blockade alone group, 265 in the steroids group, and 78 in the immunosuppression group

	Observation (*n* = 469)	RAAS blockade alone (*n* = 64)	Steroids (*n* = 302)	Other Immunosuppression (*n* = 86)	*P*-value
Urine protein available	284 (97.9)	44 (84.6)	209 (78.9)	69 (88.5)	
≥ 2 + on UA	21 (7.4)	6 (13.6)	24 (11.5)	27 (39.1)	< 0.001
UPCR at last follow up					
UPCR available	118 (28.2)	30 (57.7)	113 (42.6)	56 (71.8)	
UPCR	0.12 [0.08, 0.23]	0.24 [0.16, 0.54]	0.12 [0.07, 0.25]	0.40 [0.19, 0.96]	< 0.001
UPCR category					< 0.001
< 0.5	105 (89.0)	22 (73.3)	103 (91.2)	31 (55.4)	
0.5–2	10 (8.5)	8 (26.7)	8 (7.1)	17 (30.4)	
> 2	3 (2.5)	0 (0.0)	2 (1.8)	8 (14.3)	
Treated with RAAS blockade at last follow up	8 (1.7)	21 (32.8)	17 (5.6)	16 (18.6)	< 0.001
Hypertension diagnosis	21 (4.5)	11 (17.2)	15 (5.0)	14 (16.3)	< 0.001
Treated with antihypertensive medication at last follow up	8 (1.7)	2 (3.1)	7 (2.3)	6 (7.0)	0.038
eGFR at last follow up					
eGFR available	147 (35.2)	40 (76.9)	141 (53.2)	60 (76.9)	
eGFR	108.12 [96.78, 125.16]	103.21 [83.29, 128.44]	113.81 [92.25, 132.82]	105.71 [93.22, 126.08]	0.265
eGFR category					0.100
> 90	120 (81.6)	25 (62.5)	113 (80.1)	46 (76.7)	
≥ 60 and < 90	23 (15.6)	14 (35.0)	24 (17.0)	10 (16.7)	
≥ 30 and < 60	3 (2.0)	1 (2.5)	2 (1.4)	1 (1.7)	
< 30	1 (0.7)	0 (0.0)	2 (1.4)	3 (5.0)	
CKD stage ≥ 3	3 (2.0)	0 (0.0)	3 (2.1)	4 (6.7)	0.149
eGFR or UPCR available	199 (47.6)	44 (84.6)	175 (66.0)	64 (82.1)	
CKD ≥ 3 and/or UPCR ≥ 0.5	16 (8.0)	8 (18.2)	12 (6.9)	26 (40.6)	< 0.001
Chronic dialysis	0 (0.0)	0 (0.0)	0 (0.0)	1 (1.2)	0.021
Kidney transplant	(0.0)	0 (0.0)	0(0.0)	1(1.2)	0.021
Kidney biopsy	24 (5.1)	20 (31.2)	79 (26.2)	46 (53.5)	< 0.001
Death	0 (0.0)	0 (0.0)	0 (0.0)	0 (0.0)	NA

*UA*, urinalysis; *UPCR*, urine protein to creatinine ratio; *RAAS*, renin–angiotensin–aldosterone system; *eGFR*, estimated glomerular filtration rate; *CKD*, chronic kidney disease

## Discussion

Clinical management and outcomes in large, unselected populations of children with IgAVN have not been well-described. The present study addresses this knowledge gap in a cohort of 1139 children with IgA vasculitis who were followed by nephrology. Conservative management with observation or RAAS blockade alone was the predominant clinical practice pattern, with corticosteroids and other immunosuppressive agents reserved for patients with a more severe presentation. Outcomes were favorable among all treatment groups, with rates of advanced CKD between 0 and 6.7%.

Many studies have investigated the risk of CKD in children with IgAVN, with varying results. Significant kidney impairment has ranged from 1.2 to 21% [2, 3, 11, 17–22]. Studies including children with more moderate kidney disease (proteinuria and hypertension) have yielded long-term risk of CKD ranging from 23 to 68% [2, 6, 18, 19, 21, 22]. Most prior reports have been single-center studies, with one meta-analysis reporting long-term kidney impairment in 5.4% of children with IgAVN [21]. In this meta-analysis, however, the definition of kidney impairment involved the presence at last follow-up of either nephrotic syndrome, nephritis, kidney failure, or hypertension, precluding a more detailed estimation of specific outcomes (e.g., proteinuria, mild to moderate CKD, and KF). Accurate estimation of outcomes has further been limited by selection bias, with previous studies reporting only those undergoing kidney biopsy or with extended follow-up.

In this study, we used data from PEDSnet to provide a detailed description of the course and outcomes in children with IgAV. Overall, 29% of children in our study had kidney involvement, which is similar to rates previously reported [1, 2]. Compared to previous studies, our results showed a more favorable kidney prognosis, with only 2.6% and 0.2% of children developing CKD and KF, respectively, after a median follow-up of 1.7 years. Similarly, clinically significant proteinuria at the last follow-up was present in only 12.9% of those with urinalyses available and 17.6% among those who had UPCR measured. Our study was unique in that it focused on a cohort of all children followed by nephrology, therefore providing a more generalizable assessment of outcomes among patients managed by a pediatric nephrologist.

Several explanations may account for the lower prevalence of poor outcomes in our cohort. In contrast to previous studies, the inclusion of all patients followed by nephrology likely represents a population of children with a wide spectrum of kidney involvement. Shorter follow-up time in our cohort may also explain lower prevalence of CKD, as some previous reports encompassed a follow-up period exceeding 20 years [3, 6]. In our cohort, the prevalence of advanced CKD among those followed for more than 4 years was more than five times higher than in those with a shorter follow-up (4.7% vs. 0.9%), though still lower than some previous reports. Interestingly, among the 113 children followed for ≥ 7 years, just 1.8% had CKD stage 3 or beyond. These differences may represent progression of disease over time; conversely, patients with milder disease may simply be discharged from nephrology care. Finally, more aggressive treatment in children with severe disease, including those treated with corticosteroids and other immunosuppressive agents, may have resulted in improved outcomes.

Treatment of IgAVN remains challenging, as randomized controlled trials are lacking. To our knowledge, this is the largest study describing management patterns in IgAVN. Recent KDIGO guidelines on the management of glomerular diseases suggest RAAS inhibition as initial treatment for those with persistent proteinuria and normal kidney function [23, 24]. Monotherapy with RAAS blockade was used in only 6% of our cohort, indicating this treatment by itself is not favored among pediatric nephrologists, with observation alone or corticosteroids representing the most commonly preferred options. The overall similar and favorable outcomes among children treated with observation and RAAS blockade in our cohort suggests that conservative treatment strategies are an acceptable option in children with less severe disease.

Our study showed that immunosuppressive medications other than steroids are used in children with known risk factors for poor outcomes, including older age, decreased eGFR, and/or significant proteinuria [19, 25]. At the last follow-up, the patients managed with immunosuppressive medications continued to have more proteinuria and hypertension than those treated with steroids or conservative management. Notwithstanding these findings, kidney outcomes were good, with 6.7% progressing to CKD stage 3. This is in contrast to previous reports of children with severe disease at presentation, which report a risk of progression to CKD of 31–40% [3, 19, 22]. It may be that aggressive treatment in those with more severe diseases resulted in improved long-term outcomes. Although outcomes were similar among all treatment groups in this study, management with immunosuppressive therapy may still be warranted in children with a more severe presentation. Severity of clinical presentation and histologic lesions have both been associated with poor long-term outcomes, and therefore both should be considered in the decision to use immunosuppressive therapy [19, 25, 26]. Still, the effects of various treatment strategies on long-term risk of CKD remain unknown.

This study has several strengths that were made possible by leveraging data from the PEDSnet database. To date, this represents one of the largest studies investigating outcomes in IgAVN, including children from seven pediatric centers across the United States. In addition to hospital and billing coding, PEDSnet contains laboratory data and medication prescription history, providing information about clinical management and specific outcomes such as eGFR and proteinuria. As shown in Table 1, the demographic composition of the PEDSnet cohort is similar to that of the US population, making these results fairly generalizable. Several limitations also deserve consideration. First, our sample likely underestimated the total number of patients who actually developed any degree of IgAVN; some patients with mild nephritis may not have been seen by nephrology, as is suggested by the 12.7% with microscopic hematuria in group 0. Further, missing data (presented in tables) may have been common among patients with limited interaction with nephrology or those who were managed more conservatively. Children with normal or mild urinary findings may have followed up with clinicians outside PEDSnet institutions, or they may have not had any follow-up clinic visits or laboratory studies. As those with less severe diseases may be less likely to have consistent follow-up, outcomes reported in this study could be overestimated. Another limitation is the lack of histologic results from kidney biopsies. Histologic severity correlates with long-term outcomes and almost certainly guides decision-making in children with IgAVN. Due to the lack of histologic data and the likelihood that those children with more severe diseases were likely to receive more aggressive treatment, this study was not designed to provide a comparative analysis of the efficacy of immunosuppression in IgAVN. Prospective trials comparing treatment strategies will be required to understand the impact of specific immunosuppression on the clinical course of children with moderate to severe IgAVN.

Among children with IgAV followed by nephrologists in the PEDSnet cohort over a median 1.7-year follow-up, CKD occurred in 2.6% and KF in 0.2%. These findings indicate overall kidney outcomes may be better than previously reported when children with all severities of IgAVN are considered. Although supportive care without pharmacologic management was most frequent, immunosuppressive medications were used in those with more severe presentation and may have contributed to improved outcomes.

### Supplementary Information

Below is the link to the electronic supplementary material.

Graphical abstract (PPTX 50 KB)

## Abbreviations

BMI: Body mass index
CKD: Chronic kidney disease
DBP: Diastolic blood pressure
eGFR: Estimated glomerular filtration rate
EHR: Electronic health record
GI: Gastroenterology
HSP: Henoch-Schönlein purpura
IgAV: IgA vasculitis
IgAVN: IgA vasculitis with nephritis
IMO: Intelligent medical objects
IRB: Institutional review board
IV: Intravenous
KF: Kidney failure
MMF: Mycophenolate mofetil
PMCA: Pediatric Medical Complexity Algorithm
RAAS: Renin–angiotensin–aldosterone system
SBP: Systolic blood pressure
SMD: Standard mean difference
SNOMED-CT: Systematized Nomenclature of Medicine, Clinical Terms
UA: Urinalysis
UPCR: Urine protein to creatinine ratio
