# Supplementary material for: Clinical course and management of children with IgA vasculitis with nephritis
Source: Pediatr Nephrol. 2023 Jun 14;38(11):3721–33. doi: 10.1007/s00467-023-06023-8 (PMC10514113; doi:10.1007/s00467-023-06023-8)
Supplement: Supplementary file 1 — Graphical abstract (PPTX 50 KB) [file 467_2023_6023_MOESM1_ESM.pptx]

## Slide 1
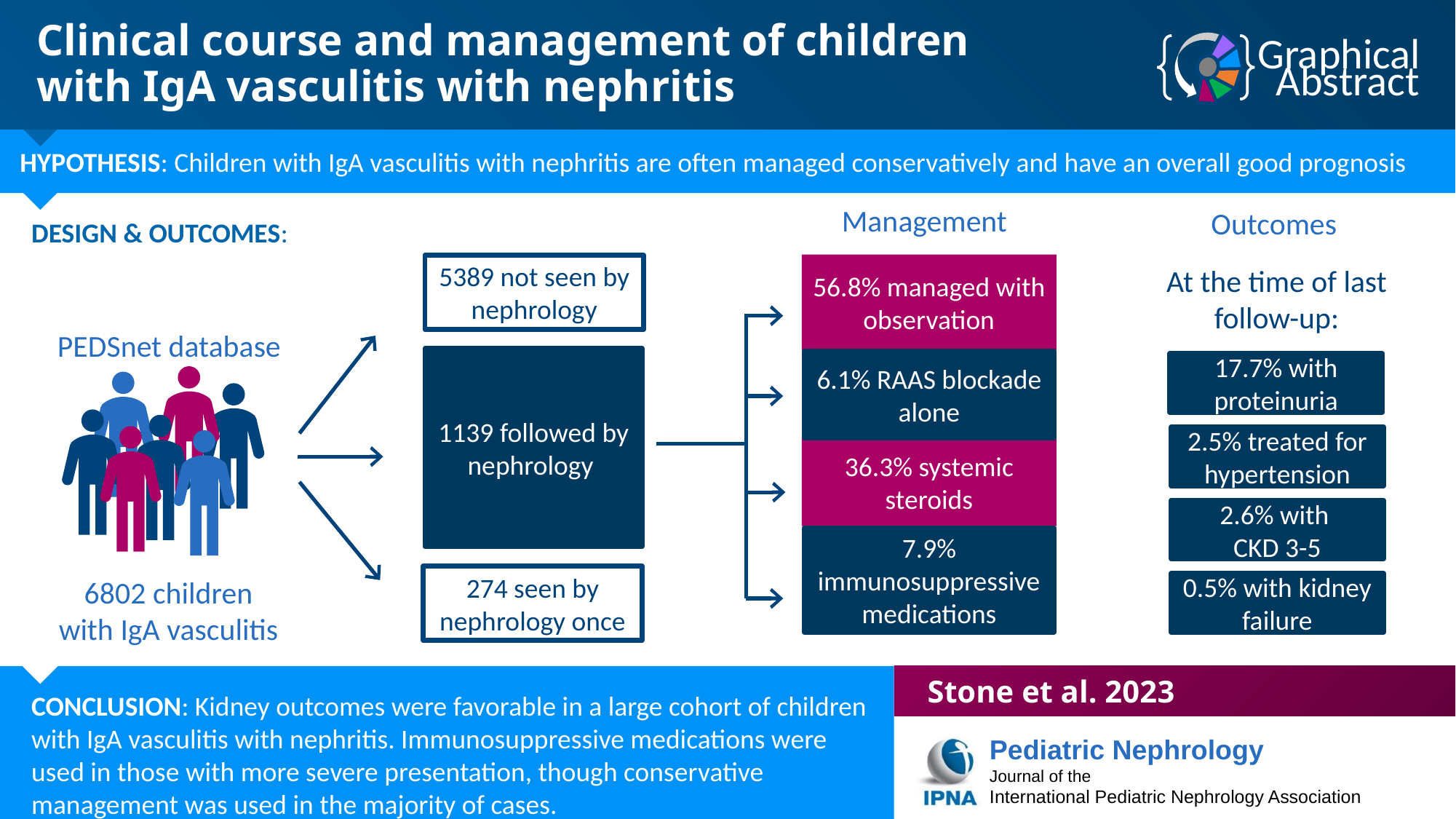

Clinical course and management of children with IgA vasculitis with nephritis
HYPOTHESIS: Children with IgA vasculitis with nephritis are often managed conservatively and have an overall good prognosis
Management
Outcomes
DESIGN & OUTCOMES:
56.8% managed with observation
6.1% RAAS blockade alone
36.3% systemic steroids
7.9% immunosuppressive medications
5389 not seen by nephrology
1139 followed by nephrology
274 seen by nephrology once
At the time of last follow-up:
PEDSnet database
6802 children with IgA vasculitis
17.7% with proteinuria
2.5% treated for hypertension
2.6% with
CKD 3-5
0.5% with kidney failure
Stone et al. 2023
CONCLUSION: Kidney outcomes were favorable in a large cohort of children with IgA vasculitis with nephritis. Immunosuppressive medications were used in those with more severe presentation, though conservative management was used in the majority of cases.
